# The French adaptation and validation of the Partners in Health (PIH) scale among patients with chronic conditions seen in primary care

**DOI:** 10.1371/journal.pone.0224191

**Published:** 2019-10-23

**Authors:** Émilie Hudon, Maud-Christine Chouinard, Cynthia Krieg, Mireille Lambert, Heithem Joober, Sharon Lawn, David Smith, Sylvie Lambert, Catherine Hudon

**Affiliations:** 1 Faculté de Médecine et des Sciences de la Santé, Université de Sherbrooke, Québec, Canada; 2 Centre intégré universitaire de Santé et de Services sociaux du Saguenay-Lac-Saint-Jean, Chicoutimi, Québec, Canada; 3 Département des sciences de la santé, Université du Québec à Chicoutimi, Chicoutimi, Québec, Canada; 4 Flinders Human Behaviour & Health Research Unit (FHBHRU), Flinders University College of Medicine and Public Health, Bedford Park, South Australia, Adelaide, Australia; 5 École des sciences infirmières, Université McGill, Montréal, Québec, Canada; 6 Département de médecine de famille et de médecine d’urgence, Université de Sherbrooke, Sherbrooke, Québec, Canada; 7 Centre de recherche du Centre hospitalier de l’Université de Sherbrooke, Québec, Canada; University of Maribor, SLOVENIA

## Abstract

**Objective:**

Measuring self-management helps identify the degree of participation of people in the management of their chronic conditions and guides clinicians in determining person-centred priorities for providing support. The *Partners in Health* scale, a self-report generic questionnaire, was developed to capture the self-management of patients with chronic conditions. This study aimed to translate the *Partners in Health* scale into French and to examine its psychometric properties in French-speaking people with chronic conditions followed in primary care.

**Methods:**

The *Partners in Health* scale was translated into French using Hawkins and Osborne’s method (2012). Content validity was evaluated through cognitive interviews (Think Aloud Method). Internal consistency was measured at baseline with Cronbach’s alpha. Test-retest reliability was evaluated at baseline and two weeks later using the intraclass correlation coefficient. Concurrent validity was measured at baseline with the Self-efficacy for Managing Chronic Disease (SEM-CD) and the Patient Activation Measure (PAM), using Spearman correlations.

**Results:**

Cognitive interviews were conducted with 10 participants. During these interviews, most items were clearly understood and accepted as formulated; only a few terms were modified. To evaluate the psychometric properties of the French-language version of the Partners in Health scale, 168 participants (male = 34.5%; mean age = 58 years; mean number of chronic conditions = 4.1) completed the questionnaire at baseline and 47 of them completed the questionnaire two weeks later by telephone. Cronbach’s alpha for internal consistency was 0.85 (95% confidence interval: 0.81–0.88). The intraclass correlation coefficient for test-retest reliability was 0.77 (95% confidence interval: 0.58–0.87). Concurrent validity with spearman’s coefficient correlation of Self-efficacy for Managing Chronic Disease and Patient Activation Measure was 0.68 and 0.61 respectively.

**Conclusion:**

The French-language version of the *Partners in Health* scale is a reliable and valid questionnaire for the measure of self-management in persons with chronic conditions seen in primary care.

## Introduction

Chronic conditions are the leading cause of mortality and are responsible for 70% of mortality rates worldwide; representing 40 million deaths per year [1]. Self-management is a complex process in which the person actively participates in the management of his or her chronic conditions. It leads to important benefits both at the individual level, such as improvement in self-control, well-being and quality of life, and at the societal level, such as a decrease in use and costs of health services [2]. Self-management involves medical/behavioral, decision-making, emotional and cognitive strategies [3, 4].

Clinicians need tools to measure self-management in order to assess the participation of the person with chronic conditions in the management of his or her health, to adjust their self-management support interventions to the needs, preferences and priorities of the person in order to maximise engagement and motivation for change, and to evaluate the impact of these interventions [5]. The Partner in Health (PIH) scale [5], developed by researchers in Australia, approximately 20 years ago, offers primary healthcare providers collaborating with people with chronic conditions a generic tool to measure the self-management of this client population. The fourth and last version of the questionnaire was published in 2016 by Smith, Harvey, Lawn, Harris and Battersby [5–8]. This version of the PIH comprises 12 items, with responses rated using a 9-point Likert-type scale. It presents a variety of interesting characteristics: 1) it targets adults with at least one chronic condition; 2) it is completed by self-report; 3) it is short (<25 items); 4) items are short (<20 words/item); 5) the psychometric properties are good; and 6) it measures all strategies of the self-management concept (behavioral/medical, cognitive/decision-making, emotional and social strategies) [3, 4, 9, 10]. Previous factor analysis identified four factors: 1) knowledge; 2) partnership in treatment; 3) recognition and management of symptoms; and 4) coping [7]. The PIH has been translated into Spanish [11], Dutch [12], and Chinese [13] and adapted for populations with diabetes [14], chronic renal disease [15, 16], mental illness [17], and for older adults with hearing loss [18]. To date, no French-language version of the PIH is available.

### Aim

The main purpose of this study was to translate the PIH into French version (PIH-Fv) and to evaluate its psychometric properties (internal consistency, test-retest reliability and concurrent validity) among a French-speaking population with at least one chronic condition and followed in primary care.

## Materials and methods

### French-language cross-cultural adaptation

The cross-cultural adaptation was conducted following the Hawkins and Osborne method (2012) [19, 20], involved the authors of the PIH, and included the following steps:

1. *Translation*: A professional native French-speaking translator translated the original version into a French version.
2. *Back-translation*: A professional native English-speaking translator back-translated the French version into English without seeing the original English version of the PIH.
3. *Committee evaluation*: An expert panel composed of researchers specialized in primary care, one developer of the PIH (SL), two translators, healthcare providers (nurses and family physicians) and one bilingual patient met to compare the original version with the back-translated version. The purpose was to clarify any inconsistencies between the two English versions and to come to an agreement in the labeling of items in French, while preserving the same meaning as the original version.
4. *Pretest*: Cognitive interviews were conducted by the first author (ÉH) with persons with various chronic conditions until data saturation was reached (n = 10). The recruitment process was the same as the one used for the validation part of this study (see next section). Interviews based on the Think Aloud Method lasted from 30 to 45 minutes [21, 22]. This consisted of having the participant read the questionnaire out loud and answering without the help of the research assistant. Any issues raised by the participants were then reviewed by the team to modify any term that lacked clarity or was confusing.
5. *Validation of the French-language version of the questionnaire*: details are provided in the methods section below.

### Settings, participants and recruitment procedures

For the evaluation of the psychometric properties of the PIH-fv, recruitment was carried out in two Family Medicine Groups from two cities located in two different regions of Québec, Canada (Saguenay-Lac-Saint-Jean and Estrie), using a convenient sampling procedure. These regions were selected to represent rural and urban areas. The inclusion criteria were: 1) being a patient in the participating clinic; 2) 18 years and older; 3) native French-language speaker; and 4) suffering from at least one chronic condition (regardless of type, time since diagnosis, or treatment). Pregnant women and patients with an acute exacerbation of their chronic condition were excluded, because these states require a more frequent follow-up that may interfere with self-management and study outcomes [23].

Recruitment was conducted May 9–26, 2016, while the patients were waiting for their appointment with a primary healthcare provider. The clinic’s receptionist provided a description of the project with a list of inclusion criteria to each patient. Two authors (EH and CK) approached patients to explain the project and assess their eligibility for participation against the inclusion criteria. Eligible persons provided consent and completed the questionnaire (T1). Two weeks later, some participants completed the questionnaire (T2) once again by telephone, without the sociodemographic and Disease Burden Morbidity Assessment (DBMA) sections. This timeframe is considered adequate to assess test-retest reliability [24]. The research protocol was approved by the ethics review board of the Centre intégré universitaire de la santé et des services sociaux. Informed consent was obtained from all individual participants in the study.

The required sample size was 120 participants, based on Polit recommendations [25]. To evaluate test-retest reliability by telephone at T2, 50 participants was deemed sufficient [26].

### Measures

The questionnaire was divided into five sections. It included a sociodemographic section (gender, date of birth, place of birth, first language, education, occupation, income and marital status), the French-language versions of the DBMA [27], the PIH, the Patient Activation Measure (PAM) [28] and the Self-efficacy for Managing Chronic Disease (SEM-CD) [29]. Because self-management is a concept associated to activation and self-efficacy as Richard and Shea [30] stated in their concept analysis, the PAM and the SEM-CD were judged to be appropriate to measure their concurrent validity with the PIH.

#### Partners in Health (PIH) scale

The PIH scale [7] is a self-report questionnaire that includes 12 items, which are answered using 9-point Likert-type scales (Items 1 and 2: 0 “very poor” to 8 “a lot”; Items 3 to 8: 0 “never” to 8 “always”; Items 9 to 12: 0 “not very well” to 8 “very well”). The total score ranges from 0 to 96, 0 representing poor self-management and 96 representing a greater self-management. Factor analysis identified four related self-management constructs, but in this study the scale was treated as unidimensional because dimensions contained less than three items and the questionnaire is considered as a whole [31] (S1 Appendix).

#### French-language version of the Patient Activation Measure (PAM)

We explored the concurrent validity of the PIH with the PAM because activation levels are significantly associated with self-management behaviors [32]. The PAM [32] is a self-report questionnaire that measures the degree of activation based on a global score transformed into a one-dimensional scale ranging from 0 to 100 (a higher score indicating higher activation). The questionnaire contains 13 items with a 4-point Likert scale (disagree strongly, disagree, agree or agree strongly). The PAM was adapted for use in French and showed good psychometric properties [28].

#### French-language version of the Self-Efficacy for Managing Chronic Disease (SEM-CD)

We also explored the concurrent validity of the PIH with the SEM-CD which measures self-efficacy, a concept also related to self-management as cited by Wilde and Garvin [33]. The SEM-CD is a self-report questionnaire measuring a person’s perception of their self-efficacy [34]. The questionnaire contains 6 items and uses an 11-point Likert scale (0–10), ranging from “not at all confident” to “totally confident”. The final score is a mean ranging from 0 to 10 of all items (a higher score indicating higher self-efficacy). The French version of the SEM-CD demonstrated good psychometric properties [29].

#### French-language version of the Disease Burden Morbidity Assessment (DBMA)

The Disease Burden Morbidity Assessment (DBMA) [35] was used to identify the number of chronic conditions, out of a total of 21. The DBMA was translated into French and demonstrated good psychometric properties [27].

### Analysis

Data were analyzed using the Statistical Package for the Social Sciences (SPSS) version 23.0 [36]. Participant characteristics, such as age and number of chronic conditions, were described using means and standard deviations (SD) for continuous variables and frequencies (%) for categorical data as presented in Table 1. Internal consistency was measured using Cronbach’s alpha (α). A Cronbach alpha between 0.70 and 0.90 indicates good internal consistency; a value below 0.70 indicates poor internal consistency, whereas above 0.90 indicates redundancy among item [37]. Test-retest reliability was measured with an interclass correlation coefficient (ICC). An ICC above 0.75 indicates good reliability, whereas an ICC below 0.75 indicates a low to moderate reliability [38]. Concurrent validity was measured with Spearman’s correlations, as data were not normally distributed.

**Table 1:** Sociodemographic characteristics of participants (n = 168).

**Age: ẋ (SD)**	**58 (15.8)**
**Number of chronic conditions: ẋ (SD)**	**4.1 (2.3)**
**Region: n (%)**	
Rural	49 (29.2)
Urban	110 (70.8)
**Male: n (%)**	**58 (34.5)**
**Chronic condition: n (%)**	
High blood pressure (HBP)	69 (42.6)
Overweight	68 (41.5)
Osteoarthritis	59 (36.2)
Depression or anxiety	59 (36.2)
**Education completed: n (%)**	
Less than high school	39 (24.1)
Completed high school	47 (29.0)
College or post-secondary school	41 (25.3)
University	35 (21.6)
**Occupation: n (%)**	
Employed	57 (34.8)
Unemployed	25 (15.2)
Retired	79 (48.2)
**Annual family income: n (%)**	
Less than $ 20 000 CAD	22 (13.5)
$ 20 000 to $ 49 999 CAD	64 (39.3)
$ 50 000 CAD or more	77 (47.2)
**Marital status: n (%)**	
Married, living with a partner	91 (54.5)
Separated, divorced	30 (18.0)
Widowed	22 (13.2)

n: sample size, SD: standard deviation, ẋ: mean, %: percentage of the population

## Results

### Translation, back-translation and committee evaluation

A French version of the PIH was obtained after comparing the original English version, the translated French version and the back-translated version. Modifications were minor, for example, the term "worse" became "worsen".

### Pretest

Cognitive interviews were conducted with 10 persons with chronic conditions. Some terms were adapted to the French language. For example, the item "I am able to deal with health professionals to get the services I need that fit with my culture, values and beliefs" was modified to "I am able to compose with health professionals to get services I need that fit with my culture, values and beliefs". Participants were not sure they understood the meaning of the term "deal", so they suggested "compose". Following the first four steps of the French translation of the PIH a French version was approved by the authors of the PIH (S2 Appendix).

### Validation of the French-language version of the questionnaire

#### Sample characteristics

**Fig 1:**
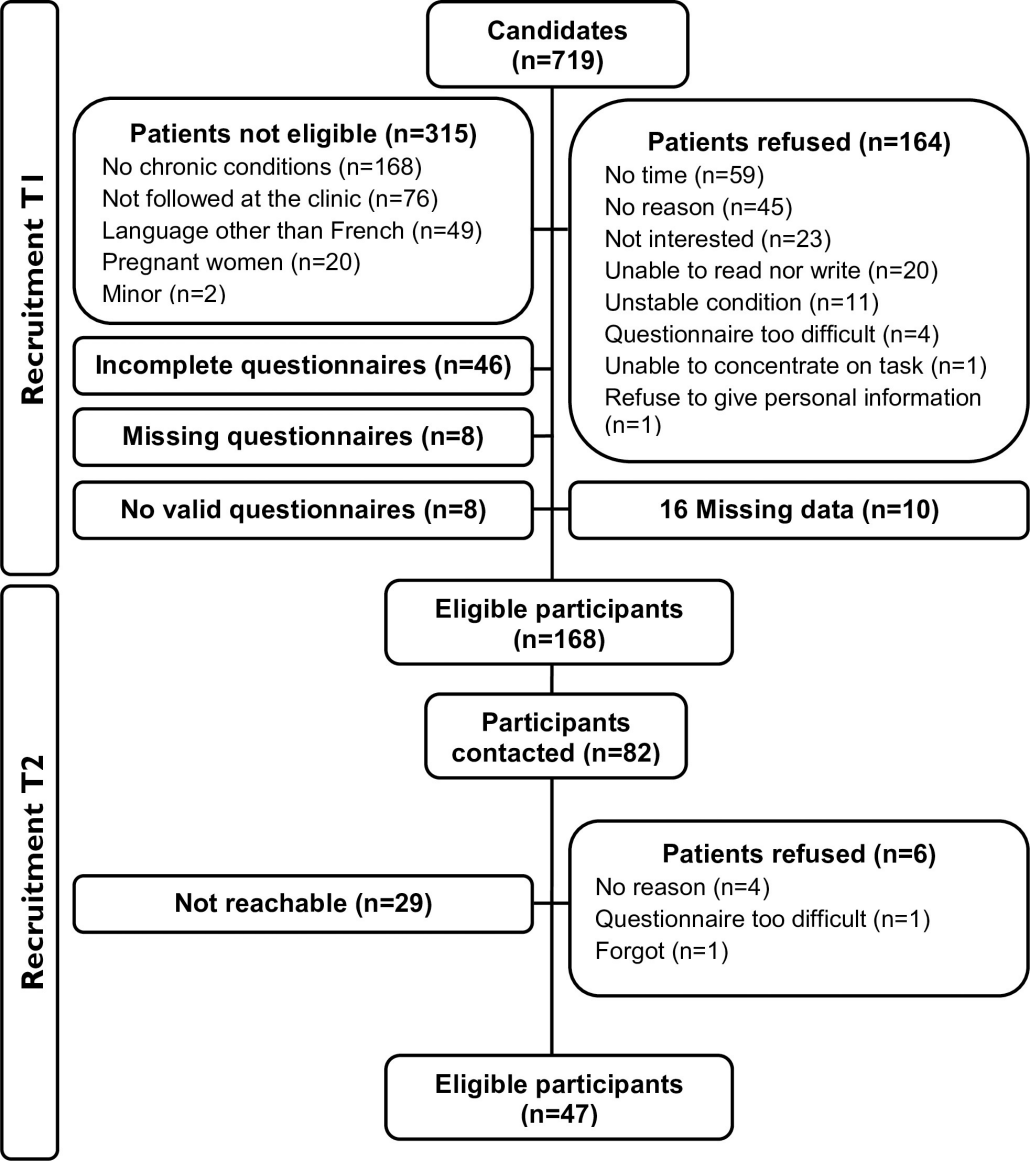
Participation in the study at T1 and T2. Definition of terms: "not eligible" represents people who did not meet selection criterions; "incomplete questionnaires" corresponds to questionnaires with an incomplete section or a section with more than half of the section missing responses; "missing questionnaires" represents the questionnaires lost during recruitment; "invalid questionnaire" represents a questionnaire completed by another person or a questionnaire with two answers to the same question; "missing data" corresponds to a questionnaire without answers at some questions; and "unreachable" represents people whose telephone was out of service when called or the people who did not answer.

For the study, 719 patients with at least one chronic condition were approached at T1 (Fig 1). Most refusals to participate (n = 164) were due to lack of time before the medical appointment. Among the 232 patients who completed the questionnaire, 46 were incomplete (less than half of the questionnaire was completed), 8 were not valid (two answers to the same question or completed by another person) and 10 questionnaires presented 16 missing data (PIH section), particularly for the item one. These questionnaires (n = 64) were excluded from the analysis. Finally, 168 participants (23.4%) completed the questionnaire at T1. At T2, 47 participants completed the questionnaire after 82 of the 168 participants of T1 were contacted (Table 1). Those samples were used for the analysis.

Participants were diagnosed with a mean average of four chronic conditions. Almost half of participants were retired (48.2%), had an annual income of $50,000 CAD (47.2%) and were married or lived with a partner (54.5%).

#### PIH analysis

Mean score at T1 was 81.1 (40.0–96.0). Cronbach’s alpha was 0.85 (95% CI: 0,81–0,88). The intraclass correlation coefficient between T1 and T2 was 0.77 (CI at 95%: 0.58–0.87). Spearman’s correlation was 0.61 with the PAM, and 0.68 with the SEM-CD.

## Discussion

The results of this study demonstrated good psychometric properties of the PIH-Fv, which are comparable to the original version and to other cross-cultural adaptations (Dutch and Chinese). To our knowledge, this is the first study of the translation and adaptation of the PIH into French. We used Hawkins and Osborne’s method. The authors of the cross-cultural adaptation of the Chinese and Dutch versions used the Brislin (1970) [39] and the Guillemin (1993) [40] methods [12, 13] respectively. These two translation methods require an evaluation committee for the translation. However, they did not mention if patients or authors of the original questionnaires participated [39, 40].

This study is also the first evaluation of the psychometric properties of the PIH-Fv. The average score for T1 (82.17) tended towards good self-management, similarly to the Dutch adaptation (ẋ = 78.1) [12]. Internal consistency of previous initial English versions [5, 6] and the Dutch adaptation [12] of the PIH yielded Cronbach’s alphas varying from 0.82 to 0.86. The internal consistency in this study remained similar after the translation process (α = 0.85). Test-retest reliability was also documented in the Chinese adaptation [13] with an ICC of 0.82, but the confidence interval was not mentioned. The coefficient (ICC = 0.77) in our study is slightly lower than the Chinese adaptation. In our, study the ICC could have been affected by a medical visit between T1 and T2 [38] which might have provided some self-management support explaining an increase in total score (81.1 at T1 versus 84.7 at T2). This result may also be linked to the change in the administration procedure of the PIH-Fv at T1 (in person) and T2 (by telephone) [41].

This is the first study to measure the concurrent validity of the PIH with the SEM-CD and the PAM. The concurrent validity of the PIH-Fv measuring self-management showed a moderate correlation with the concept of activation as measured by the PAM and with the concept of self-efficacy as measured by SEM-CD. This is explained by the fact that these concepts are related [9].

### Strengths and limitations

#### Strengths

The study was conducted in two different regions of Quebec, which covers a rural population (n = 49) as well as an urban one (n = 119), making the sample more representative of the population.

The study was performed rigorously, using Hawkins and Osborne’s method, which allowed us to conduct the cross-cultural French adaptation of the questionnaire while preserving the original meaning of the questions by consulting the authors of the PIH. Finally, all information collected and entered into the database was validated by a second person, independently, ensuring accuracy.

#### Limitations

Participants had to complete the questionnaire while sitting in the clinics’ waiting rooms. If the patient’s appointment with the clinician was ahead of time and the participants did not have enough time to complete the questionnaire, it was rarely completed after that appointment. This may explain the high number of incomplete questionnaires (n = 45).

To further appraise PIH-Fv psychometric properties, a next study should recruit a larger sample to evaluate PIH-Fv structural validity. In addition, it may be appropriate to conduct a sensitivity to change analysis in a clinical intervention study to assess the capacity of the PIH-Fv to capture clinical improvement of self-management level [7]. Finally, missing data were particularly associated with item one of the PIH-Fv. This could be explained by the configuration of the questionnaire; the first question was presented directly below examples of answer options. To reduce the number of missing data, it would be preferable to separate examples of answers options more clearly at the beginning to the PIH-FV. Finally, we suggest conducting a pre-test to evaluate cultural differences before using the French version with other French populations.

## Conclusions

The PIH-Fv demonstrated good internal consistency, good test-retest reliability and moderate concurrent validity with the PAM and the SEM-CD. The PIH-Fv can be used to measure level of self-management among French-speaking adults with chronic conditions.

## Supporting information

S1 Appendix Factors and items of the PIH questionnaire. PIH with a 9-point Likert scale; 12 questions assessing four factors: Knowledge (items 1–2), partnership in treatment (items 3–6), recognition and management of symptoms (items 7–8) and coping (items 9–12). Licence tool: +61 8 8404 2607. For more information’s contact original authors at: ccm@flinders.edu.au.
(DOCX)

S2 Appendix Factors and items of the PIH-Fv questionnaire. French-language version of the PIH with a 9-point Likert scale; 12 questions assessing four factors: Knowledge (items 1–2), partnership in treatment (items 3–6), recognition and management of symptoms (items 7–8) and coping (items 9–12).
(DOCX)
