# Supplementary material for: The French adaptation and validation of the Partners in Health (PIH) scale among patients with chronic conditions seen in primary care
Source: PLoS One. 2019 Oct 23;14(10):e0224191. doi: 10.1371/journal.pone.0224191 (PMC6808494; doi:10.1371/journal.pone.0224191)
Supplement: S1 Appendix — PIH with a 9-point Likert scale; 12 questions assessing four factors: Knowledge (items 1–2), partnership in treatment (items 3–6), recognition and management of symptoms (items 7–8) and coping (items 9–12). Licence tool: +61 8 8404 2607. For more information’s contact original authors at: ccm@flinders.edu.au. (DOCX) [file pone.0224191.s001.docx]

**S1 Appendix. Factors and items of the PIH questionnaire**

| **Item** | **Likert Scale** |  |  |
| --- | --- | --- | --- |
| **Factor 1: Knowledge** | | |  |
| 1. Overall, what I know about my health condition is: | \| 0 1 2 3 4 5 6 7 8 \| \| --- \| \| Very little Something A lot \| | | |
| 1. Overall, what I know about my treatment, including medications for my health condition(s) is: | \| 0 1 2 3 4 5 6 7 8 \| \| --- \| \| Very little Something A lot \| | | |
| **Factor 2: Partnership in treatment** | | |  |
| 1. I take medications or carry out the treatments asked by my doctor or health worker: | \| 0 1 2 3 4 5 6 7 8 \| \| --- \| \| Never Sometimes Always \| | | |
| 1. I share decisions made about my health condition(s) with my doctor or health worker: | \| 0 1 2 3 4 5 6 7 8 \| \| --- \| \| Never Sometimes Always \| | | |
| 1. I am able to deal with health professionals to get the services I need that fit with my culture, values and beliefs: | \| 0 1 2 3 4 5 6 7 8 \| \| --- \| \| Never Sometimes Always \| | | |
| 1. I attend appointments as asked by my doctor or health worker: | \| 0 1 2 3 4 5 6 7 8 \| \| --- \| \| Never Sometimes Always \| | | |
| **Factor 3: Recogition and management of symptoms** | | |  |
| 1. I keep track of my symptoms and early warning signs (e.g. blood sugar levels, peak flow, weight, shortness of breath, pain, sleep problems, mood): | \| 0 1 2 3 4 5 6 7 8 \| \| --- \| \| Never Sometimes Always \| |  |  |
| 1. I take action when my early warning signs and symptoms get worse: | \| 0 1 2 3 4 5 6 7 8 \| \| --- \| \| Never Sometimes Always \| |  |  |
| **Factor 4: Coping** | | |  |
| 1. I manage the effect of my health condition(s) on my physical activity (i.e. walking, household tasks): | \| 0 1 2 3 4 5 6 7 8 \| \| --- \| \| Not very well Fairly well Very well \| |  |  |
| 1. I manage the effect of my health condition(s) on how I feel (i.e. my emotions and spiritual well-being): | \| 0 1 2 3 4 5 6 7 8 \| \| --- \| \| Not very well Fairly well Very well \| |  |  |
| 1. I manage the effect of my health condition(s) on my social life (i.e. how I mix with other people): | \| 0 1 2 3 4 5 6 7 8 \| \| --- \| \| Not very well Fairly well Very well \| |  |  |
| 1. Overall, I manage to live a healthy life (e.g. no smoking, moderate alcohol, healthy food, regular physical activity, manage stress): | \| 0 1 2 3 4 5 6 7 8 \| \| --- \| \| Not very well Fairly well Very well \| |  |  |

Legend S1 Appendix. PIH with a 9-point Likert scale; 12 questions assessing four factors: Knowledge (items 1-2), partnership in treatment (items 3-6), recognition and management of symptoms (items 7-8) and coping (items 9-12).

Licence tool: +61 8 8404 2607. For more information’s contact original authors at: [ccm@flinders.edu.au](mailto:ccm@flinders.edu.au)
