# Supplementary material for: The French adaptation and validation of the Partners in Health (PIH) scale among patients with chronic conditions seen in primary care
Source: PLoS One. 2019 Oct 23;14(10):e0224191. doi: 10.1371/journal.pone.0224191 (PMC6808494; doi:10.1371/journal.pone.0224191)
Supplement: S2 Appendix — French-language version of the PIH with a 9-point Likert scale; 12 questions assessing four factors: Knowledge (items 1–2), partnership in treatment (items 3–6), recognition and management of symptoms (items 7–8) and coping (items 9–12). (DOCX) [file pone.0224191.s002.docx]

**S2 Appendix. Factors and items of the PIH-Fv questionnaire**

| **Item** | **Likert Scale** |  |  |
| --- | --- | --- | --- |
| **Facteur 1 : Connaissances** | | |  |
| 1. En général, mes connaissances au sujet de ma condition de santé sont : | \| 0 1 2 3 4 5 6 7 8 \| \| --- \| \| Très faible Moyenne Très bonne \| | | |
| 1. En général, mes connaissances des traitements de ma condition de santé, incluant la médication, sont : | \| 0 1 2 3 4 5 6 7 8 \| \| --- \| \| Très faible Moyenne Très bonne \| | | |
| **Facteur 2 : Collaboration dans les traitements** | | |  |
| 1. Je prends la médication ou je suis le traitement recommandé par mon médecin /professionnel de santé : | \| 0 1 2 3 4 5 6 7 8 \| \| --- \| \| Jamais Quelquefois Toujours \| | | |
| 1. Je participe aux décisions prises au sujet de ma condition de santé avec mon médecin ou professionnel de santé : | \| 0 1 2 3 4 5 6 7 8 \| \| --- \| \| Jamais Quelquefois Toujours \| | | |
| 1. Je suis capable de composer avec les professionnels de la santé afin d’obtenir les services dont j’ai besoin qui correspondent à ma culture, mes valeurs et mes croyances : | \| 0 1 2 3 4 5 6 7 8 \| \| --- \| \| Jamais Quelquefois Toujours \| | | |
| 1. Je me présente aux rendez-vous tel que demandé par mon médecin ou professionnel de santé : | \| 0 1 2 3 4 5 6 7 8 \| \| --- \| \| Jamais Quelquefois Toujours \| | | |
| **Facteur 3 : Reconnaissance et gestion des symptômes** | | |  |
| 1. Je suis capable de reconnaître mes symptômes et signaux d’alarmes (p. ex. taux de sucre dans le sang, poids, souffle court, douleur, problèmes de sommeil, humeur) : | \| 0 1 2 3 4 5 6 7 8 \| \| --- \| \| Jamais Quelquefois Toujours \| |  |  |
| 1. J’agis quand mes symptômes et mes signaux d’alarmes s’aggravent : | \| 0 1 2 3 4 5 6 7 8 \| \| --- \| \| Jamais Quelquefois Toujours \| |  |  |
| **Facteur 4 : Vivre avec** | | |  |
| 1. Je gère les conséquences de ma condition de santé sur mes activités (c’est-à-dire marcher, tâches ménagères) : | \| 0 1 2 3 4 5 6 7 8 \| \| --- \| \| Pas très bien Plutôt bien Très bien \| |  |  |
| 1. Je gère les conséquences de ma condition de santé sur la manière dont je me sens (c’est-à-dire mes émotions et mon bien-être spirituel) : | \| 0 1 2 3 4 5 6 7 8 \| \| --- \| \| Pas très bien Plutôt bien Très bien \| |  |  |
| 1. Je gère les conséquences de ma condition de santé sur ma vie sociale (c’est-à-dire les relations que j’ai avec d’autres personnes) : | \| 0 1 2 3 4 5 6 7 8 \| \| --- \| \| Pas très bien Plutôt bien Très bien \| |  |  |
| 1. En général, je mène une vie saine (p. ex.: sans tabac, consommation d’alcool modérée, alimentation saine, activité physique régulière, bonne gestion du stress) : | \| 0 1 2 3 4 5 6 7 8 \| \| --- \| \| Pas très bien Plutôt bien Très bien \| |  |  |

Legend S2 Appendix. French-language version of the PIH with a 9-point Likert scale; 12 questions assessing four factors: Knowledge (items 1-2), partnership in treatment (items 3-6), recognition and management of symptoms (items 7-8) and coping (items 9-12)
